# Environmental Justice and Indigenous Environmental Justice

**DOI:** 10.1007/978-3-030-61071-5_2

**Published:** 2021-02-16

**Authors:** Meg Parsons, Karen Fisher, Roa Petra Crease

**Affiliations:** School of Environment, University of Auckland, Auckland, New Zealand

## Abstract

In this chapter we provide a broad overview of three dominant ways environmental justice is framed within the scholarship and consider how Indigenous peoples’ understanding and demands for environmental justice necessitate a decolonising approach. Despite critiques, many scholars and policymakers still conceive of environment justice through a singular approach (as distributive equity, procedural inclusion, or recognition of cultural difference). Such a narrow reading fails to appreciate the intersecting and interacting processes that underpin environmental (in)justices faced by Indigenous peoples. We argue that the theoretical discussions and empirical research into environmental (in)justice need to extend beyond Western liberal philosophies and instead consider pluralistic approach to Indigenous environment justice which is founded on Indigenous ontologies and epistemologies, which include intergenerational and more-human-human justice requirements.

In this chapter, we outline the four essential ideas or proposals that provide the theoretical framework of this book. Firstly, the dominant framings and articulations of environmental justice (EJ) do not account for the complexities of Indigenous intergenerational environmental justice. Secondly, scholars and decision-makers need to consider what EJ is and how it can be taken into account in the context of environmental governance and management that goes beyond a narrow framing of justice as distributive equity, procedural inclusion, or recognition of Indigenous rights and consider the intersecting and interacting processes that underpin environmental (in)justices faced by Indigenous peoples. Thirdly, the theoretical discussion of EJ needs to recognise Indigenous sovereignties, cultures, and identities through Indigenous ontologies and epistemologies rather than through Western liberal thought and governance approaches. And lastly, the theoretical underpinnings of the study of Indigenous environmental justice (IEJ) need to incorporate intergenerational considerations.

These four ideas or arguments allow us to consider and explore the theoretical and empirical gaps within the literature on EJ. Besides, it provides us space to explore how a diversity of different scholars (Indigenous and Indigenous allies) from a wide array of fields (human geography, political science, sociology, anthropology, history, Indigenous studies, environmental management, economics, philosophy, climate change adaptation) are calling for pluralistic accounts of justice that take into account local contexts, legal orders, and ontologies. Indeed, EJ always existed in the complex, overarching framework that was interwoven with the goals of social justice. As Taylor (2000) argues, in the USA context, the social injustices experienced by African-Americans and Indigenous Nations (slavery, discrimination, genocide, land confiscation) were also types of environmental injustice; policies and practices that resulted in social injustices also influence how communities were able to engage with environments and access resources (Taylor 2000). Accordingly, it is vital to highlight the ways EJ as a field of academic study and movement lassos the environmental and social together, particularly in the context of Indigenous EJ (as we will demonstrate later in this book through our case study of the Waipā River).

## EJ: Distributive Justice

Early EJ research employed a distributive justice lens to examine the inequitable distribution of environmental risks and the physical proximity of specific communities to the environmental risk (Walker 2009). EJ (EJ) scholar trace origins of EJ (as a movement and a field of study) to Warren County (North Carolina USA) where a hazardous waste storage site (Polychlorinated Biphenyl PCB) was established near low-income Black communities despite widespread community protests. A wealth of subsequent different studies, beginning with Warren County, elsewhere in the USA and around the globe, investigated the differential exposure of communities to hazardous and toxic facilities (Bevc et al. 2007; Bullard 1993; Burwell and Cole 2007; Greife et al. 2017; Pastor et al. 2001; Wilson et al. 2012). These studies widely found, in a diversity of local and national contexts, that marginalised populations (ethnic minority groups, low-income, lower-caste, undocumented migrants, Indigenous peoples) were significantly more likely to live near environmental risks than the privileged populations (an ethnic majority, high-income, higher-caste, homeowners, citizens, settlers) (Arcury and Quandt 2009; Gordon et al. 2010; Salazar 2009; Salazar and Alper 2011; Vickery and Hunter 2016). The focus in these studies was on the distribution of environmental risks (or the environmental “bads” or negative impacts of environmental hazards) across populations and geographical areas using statistical and spatial analyses (Bell and Ebisu 2012; Fisher et al. 2006; Kingham et al. 2007; Pearce et al. 2006). Later research expanded beyond just the placements of environmental risks (such as polluted waters and contaminated soils) and examined where environmental “goods” or positives (such as clean water and land) was located (Caney 2008; Holifield et al. 2017).

In the case of Warren Country scholars declare it an example of environmental racism, referring to intentional, overt, and malicious acts of EJ against specific ethnic groups (specifically non-White) (Figueroa 2001; Pulido 2017). Scholar Bullard argues that such environmental racism was (and still is) widespread in the context of the USA (Bullard 2002). Indeed, as the work of other scholars attests to, racism remains a persistent feature of environmental governance, management, planning, and decision-making processes in many different colonial contexts, including the settler nation of Aotearoa.

EJ scholars argue that racism plays a critical factor in environmental planning and decision-making processes in the US and other settler nations. In Aotearoa few academic studies explicitly examine the distribution of environmental injustices across populations and areas (Coombes 2013; Pearce and Kingham 2008; Pearce et al. 2011; Rixecker and Tipene-Matua 2003); one study found that 40 per cent of low-income neighbourhoods in Wellington were exposed to environmental harms compared to 10 per cent of high-income areas (Salmond 1999). However, Māori activists and community leaders frequently speak out about issues pertinent to discussions of environmental racism; environmental harms (pollution generating factories, hazardous waste disposal sites, contaminated lands and waters) are frequently being located in poorer non-Pākehā (chiefly Māori and Pasifika) neighbourhoods. Greensill (2010) cites the example of the town of Kawerau situated in the Bay of Plenty near where the lead author (Parsons) grew (Greensill 2010). The population of Kawerau (according to the 2018 census) was 7146 people of whom 62 per cent identified as Māori; Kawerau is one of the only three areas in contemporary Aotearoa with a Māori-majority populace (the others being Ōpōtiki and Wairoa). The town is the site of Aotearoa’s largest paper mill, established in 1953, which generates substantive pollutants (released into the air and water and deposited onto land). In particular, the mill discharges a toxic mixture of wastewater and solid materials directly into the Tarawera River. Dubbed the “Black Drain”, the local iwi (Ngāti Rangitihi) reported that they could no longer collect customary food sources (fish, watercress, and birds) due to biodiversity loss as well as health risks associated with eating contaminated foods from the river, similarly, they no longer swam in the river due to the danger it posed to their health (Davison 2009; Dodd 2010). Also, certain types of millworkers (particularly those involved in processing tasks) are more likely to be exposed to toxic chemicals during daily; processing jobs (lower-paid and supposedly lower-skilled) are overwhelming held by Māori, whereas managerial roles (higher-paid and supposedly higher-skilled) are held by Pākehā. Accordingly, the Kawerau example could be read as an example of environmental racism and the inequitable distribution of environmental harms (however, further in-depth studies are needed).

A wealth of scholars now critiqued early EJ research for framing of EJ solely in terms of distributive equity (Mills 2015; Schlosberg 2003, 2004). Distributive justice is based on the assumption that if everyone is given equal access to environmental goods and balanced exposure to environmental harms then no environmental injustice occurs (Schlosberg 2004) So, for example, if a toxic waste dump is located an equal distance from Indigenous and White communities then there would be no environmental injustice accordingly to this framing of EJ as distributive justice (Sze and London 2008). However, such a framing of EJ ignores the social, cultural, and institutional contexts in which environmental injustices take place and the historical and contemporary systematic acts of discrimination against marginalised populations (including Indigenous peoples and other non-White non-Indigenous communities in settler-colonial societies, members of lower-incomes and lower-castes in India, and formerly colonised peoples throughout the Global South) which all play substantial roles in creating environmental injustices. A distributive-framing of EJ (along with environmental racism) therefore misses crucial opportunities to critique the parts of colonialism and capitalism in its relation to different subjectivities and how it creates place-based and culturally-situated environmental injustices (Hendlin 2019; Jackson 2018; Swyngedouw and Heynen 2003; Whyte 2014, 2016a). More recent EJ scholarship demonstrates that the narrow focus on equitable distribution largely ignores the broader social, cultural, and institutional contexts in which environmental injustices take place and the role that capitalism, colonialism, and patriarchy plays in legitimising, driving and deepening environmental inequities (Álvarez and Coolsaet 2018; McGregor 2015; Swyngedouw and Heynen 2003; Sze and London 2008; Walker 2009). Later EJ research draws attention to the need to consider how procedures (policies, decision-making processes, and participation) and recognition (of cultural differences) play in EJ.

## Procedural Justice

Scholars draw attention to the need to consider procedural justice to combat the issues associated with distributive justice. Procedural-based EJ focuses on decisions and the decision-makers involved with environmental management decisions. In early EJ research, there was an unspoken assumption that the decision-makers where institutions of power (for example government agencies and energy companies) with communities (mostly poor and non-white communities) as the helpless victims of these decisions (Antadze 2018; Pitea 2009). Walker notes in several works that agencies such as the Environmental Protection Agency (US) developed policies and procedures to facilitate community input in decision making and hold guilty agencies responsible. However, as the works of Banisar et al. (2011) and others argued that these spaces of public participation, in the form of submissions, and public ways, did not yield the outcomes that communities hoped for (Banisar et al. 2011; Paloniemi et al. 2015). Often these spaces were controlled by either government agencies or the companies themselves, who were committed to focusing on their agendas rather than on community needs. This reflects a broader scholarship on public participation in environmental management, informed by the work of Arnstein, Tritter and McCallum, who argue that the majority of government attempts to include the public (or specific social groups) are superficial, and there remain considerable constraints on communities capacities to participate in the decision-making process (Arnstein 1969; Tritter and McCallum 2006).

A wealth of scholarship exploring public participation in environmental management and EJ builds on the seminal work of Arnstein, specifically the article “A Ladder of Citizen Participation” (Arnstein 1969; Boone and Buckley 2017; Carpentier 2016; Connor 1988; Hurlbert and Gupta 2015; Ross et al. 2002). Arnstein (1969) argues that participation is the “cornerstone” of democracy; however, marginalised communities demand a form of involvement that goes beyond simply just being consulted about decisions and be involved in and shape the decisions. Such participation, as Arnstein notes, calls for a redistribution of power (from the powerful to the marginalised groups within society) to enable those who are marginalised to join the conversation to determine how information is shared and ultimately encourage social reform that allows previously marginalised communities to benefit (Arnstein 1969). Arnstein breaks down participation into a ladder which is broken up into eight different steps. The steps are then grouped into three categories (Non-participation, Degrees of Tokenism and Degrees of Citizen Power); which range from the no or little public participation in decision-making (Non-participation) to some public participation (Degrees of Tokenism), and finally, a significant amount of participation and the capacities to shape government decisions (Degrees of Citizen Power).

Arnstein’s participation ladder is not without criticism amongst scholars (Carpentier 2016; Hart 2008). Indeed, those in positions of power in a society are often highly resistant to giving up any power and, as the work of feminist and anti-racist scholars demonstrates, the continuation of patriarchal structures as well as racism and other discriminatory beliefs effectively set up roadblocks to specific groups’ achieving higher levels of participation (what Arnstein terms “Degrees of Citizen Power”) in decision-making (Azmanova 2012; Crease et al. 2019; Pulido and De Lara 2018; Schlosberg 2003; Sen 1995; Tschakert and Machado 2012). The roadblocks for marginalised social groups being able to participate in environmental governance and management, as our later analysis of co-governance arrangements for the Waipā River (Chap. 10.1007/978-3-030-61071-5_7), include lack of access to appropriate financial resources, technologies, and training, as well as public participation forums being designed to fit the intellectual, cultural, and political traditions of the dominant social group (and in doing so re-articulating the state’s exclusion of Indigenous knowledge, values, and practices). Thus, it is not just limited resources and capacities that create barriers to marginalised groups participating in environmental management decision-making processes; it is also the failure of the state to recognise different cultures’ values, knowledges, and ways of life. Indeed, as Blue et al. (2019) recently highlights, participatory practices and justice are closely related (Blue et al. 2019) and, as the work of Nancy Fraser also demonstrates (Fraser 1990, 1995, 2007, 2009), people’s abilities to participate in decision-making processes are influenced by a range of economic, political and socio-economic factors that extend beyond distributive and procedural and also include recognition of cultural differences.

## Recognition Justice

Other scholars advocate for thinking about EJ as recognition and respect of individual and communal cultural differences (Barnhill-Dilling et al. 2020; Fraser 1995). Particularly in the context of water security, ecosystem restoration, and biodiversity conservation, recent scholarship examines the discursive and practical constraints of the dominant Western liberal framings of distributive and procedural EJ (He and Sikor 2015; Martin et al. 2016; Sikor et al. 2014; Sze 2018). Instead, Martin (2016), Sze (2018) and other scholars (Barnhill-Dilling et al. 2020) argue that recognition is a critical part of justice and a “necessary precondition for participating in environmental decisions” (Barnhill-Dilling et al. 2020, p. 84).

A lack of recognition, Schlosberg (2004) and Adger et al. (2011), of the impacts of environmental degradation and risks faced by specific communities, can detrimentally affect both the material and cultural wellbeing of individuals and communities’ (Adger et al. 2011; Schlosberg 2012; Schlosberg and Carruthers 2010). If, for instance, national or local governments do not acknowledge that existence of specific environmental harms, hazards, or risks (be it the pollution of waterways or the impacts of climate change) that are occurring within their jurisdictions, they are likely to be apathetic to the environmental risks and take limited actions to mitigate those risks. Similarly, if governments, interest groups, leaders, and the citizenry as a whole do not recognise that marginalised populations, including Indigenous peoples, (within their nation-states and around the world) are the most at risk (most vulnerable) to the negative impacts of environmental hazards (including water pollution, a tropical cyclone or the effects of climate change), resources are unlikely to be directed at assisting those groups (Rydin 2006; Schlosberg and Collins 2014). Hurricane Katrina, which devastated the US city of New Orleans in 2005, is a glaring example of this and is widely analysed by justice scholars. In New Orleans, a natural hazard was transformed into a disaster when distributive injustices ( environmental racism against the Black population) coincided with procedural and recognitional injustices (inequitable institutional arrangements, planning regimes, legal systems and economic structures) to marginalise the lives, bodies, and ways of life of individual people (Black/African-American residents) over others based on race (White residents). The hurricane became a large-scale disaster and was a consequence of flood levees failing and flooding predominately Black neighbourhoods, resulting in the deaths of more than 1800 people (the majority of whom were Black). Yet, scholars concur that these deaths were mostly avoidable and a consequence of actions to address the multiple social and environmental injustices faced by Black communities in New Orleans (Bullard and Wright 2008; Miller and Rivera 2009; Rohland 2018).

Scholar highlight how the settler state’s failure (or misrecognition) of Indigenous communities (by marginalising their knowledge, values, ways of life and excluding it from decisions) contributes to environmental injustices (Barnhill-Dilling et al. 2020; Holifield 2012; Holifield et al. 2017). Examples of misrecognition extend beyond the misrecognition of the culture and includes the misrecognition of land and water (and Indigenous people’s relationships with their properties, waters, and biota). Such misrecognition of lands consists of the common practice whereby settler nation-states (and settlers) devalued indigenous lands, labelling it ‘wastelands’, ‘unusable’, or ‘undesirable’ (until the land was no longer held by Indigenous peoples). These labels make it easier for settler state, settlers, and companies to justify the placement of environmental harms or risks in undesirable or marginal lands (Barnhill-Dilling et al. 2020; Holifield 2012; Walker 2009). Misrecognition, however, is only one part of the framing of EJ as recognition. The other part is recognitional justice, which is critical for Indigenous people, are the capacities of people to determine their interpretation of what environmental (in)justice is (Jackson 2008; Lowitt et al. 2019; Whyte 2011). Indeed, for Indigenous peoples who already possess or want treaties and laws that acknowledge and enforce their self-determination rights and tribal sovereignty. Even when settler states recognise indigenous peoples’ rights of self-determination, their capacities to make decisions and enact their sovereignty are often under-minded by the settler state and other outside organisations (Holifield 2012; Ranco 2008). While most scholars agree that both procedural justice and justice as recognition are essential to EJ, many scholars also declared that procedural justice and recognition alone do not provide enough to guarantee EJ.

Recognition can consist of an affirmation of a group’s cultural difference and identity and/or strategies that are directed at overcoming institutional harms that prevent meaningful engagement with political and social institutions. Recognition-informed actions include those that aim to address or mitigate injustices against Indigenous peoples through strategies termed affirmation actions (such as educational scholarships and provision of welfare). Through projects that aim to transform Indigenous-non-Indigenous relationships (such redistribution of the benefits and altering modes of production), recognition approaches are primarily directed at social and cultural changes including the “deconstruction” of principal arrangements of socio-cultural representation in ways that recognise and “change everyone’s social identities” (Fraser and Honneth 2003, pp. 12–13).

## Critique of Recognition

Dene political theorist Coulthard (2007, 2014), writing in the context of settler-colonial Canada and Indigenous nations, critiques the idea that the relationships between the settler-nation and Indigenous peoples are transformed through the “politics of recognition” (2007, p. 438). Recognition, Coulthard defines in terms of to the affirmative acknowledgement “of societal, cultural differences” and “freedom and wellbeing of marginalised individuals and groups living in ethnically diverse states” (Coulthard 2007, p. 438). Coulthard maintains that recognition-based conceptualisations of justice, emerging from Western liberal pluralism, aim to reconcile Indigenous sovereignty claims (which range from complete nation-state sovereignty to limited self-determination) with the sovereignty of the nation-state through a compromise of sorts. The state recognises Indigenous cultural identities and engages in projects aimed at improving and reconfiguring the relationships of Indigenous peoples with the nation-state. Coulthard (2007) notes that the “politics of recognition” in its present form simply reproduces “the very configurations of colonial power that Indigenous peoples’ demands for recognition have historically sought to transcend” (Coulthard 2007, p. 439). Indeed, Coulthard (2014) observes that despite different Indigenous peoples in Canada achieving recognition through legislation, Treaties, and other formal agreements with the federal and provincial governments, the Canadian courts continue to declare that the settler-state possess the right to make decisions about environmental management and developments within Indigenous landscapes and waterscapes. The vast majority of government-sponsored projects, including the construction of infrastructure and settlements as well as hydroelectric, forestry, agricultural and mining ventures, is justified and rationalised so long as each project is “‘consistent with the special fiduciary relationship’ between the Canadian government and the indigenous peoples” (Coulthard 2007, p. 451).

Other academics, following on from the work of Coulthard, similarly demonstrate how existing neoliberalism (in Aotearoa, Australia, Canada and beyond) has influenced and constrained the forms of recognition proposed by the state as a method to address social injustices experienced by Indigenous peoples as a consequence of settler colonialism (Azmanova 2012; Bargh 2018; Bell 2018; McCormack 2018). Avril Bell, for instance, highlights how:

At its Hegelian roots, recognition theory is about the struggle to achieve a relationship of equals between two subjects. To recognise the subjectivity of another is to recognise their equal and autonomous status as self-determining people worthy of respect. (Bell 2018)

What prevails is (in the words of Jakeet Singh) “recognition from above” in which the state “is the arbiter of just and unjust claims for recognition from subordinate groups” (Singh 2014a, p. 47). Aside from deciding what types of recognition are on offer, the state also spells out the provisions of recognition. For instance, while the state may legally acknowledge Indigenous rights and identities, as a range of critical humanities and social science scholars demonstrate, those rights and identity are frequently essentialised in ways that enable the state’s economic interests in the era of neoliberalism (Bargh 2018; Coombes et al. 2012; Coulthard 2014; Singh 2014b, 2019).

Avril Bell’s examination of how local governments in Aotearoa recognise Māori provides a sharp critique of how neoliberal politics influenced and constrained the form of recognition on offer by the state (Bell 2018). She highlights how the central government of Aotearoa (the Crown) now officially recognises that Māori and the Crown are Treaty partners (as encapsulated in Aotearoa’s founding document Te Tiriti o Waitangi/the Treaty of Waitangi), the legislation governing local government explicitly states that local authorities are not the Crown and are not Treaty partners with Māori. Since environmental governance is highly devoted in Aotearoa, the failure to legally include local government as Treaty partners means that local government authorities routinely misrecognise Māori interests, only allow for Māori participation in planning that is tokenism, and make no attempts to achieve distributive equity (Bell 2018; Ryks et al. 2010). Accordingly, local government is, in Bell’s view, emblematic of the failure of the New Zealand Crown to adequately recognise Māori as full Treaty partners (which we discuss further in Chap. 10.1007/978-3-030-61071-5_2) (Bell 2018). We will pick up on Bell’s analysis further in our review of the management of freshwater within the Waipā River catchment (Chaps. 10.1007/978-3-030-61071-5_4, 10.1007/978-3-030-61071-5_5, and 10.1007/978-3-030-61071-5_6) and highlight how the current politics of recognition, within the context of freshwater management, does not challenge the settler state to reform itself. Indeed, we echo Bell’s argument that when the two arms of government (central and local) are assessed together, the settler state is “not a fit subject for recognition politics” (that is to impose “recognition from above”) (Bell 2018, p. 78). At the local level, the state suffers from ongoing historical amnesia (continuing to frame local histories as one of peaceful settlement and continuous progress) and, more generally, makes endless statements that emphasise the rights of Māori iwi (as Treaty partners and as tribal authority-holders referred to as mana whenua); the importance of incorporating mātauranga (Māori knowledge) and tikanga (laws and principles) into freshwater management, yet at the same time taking actions that are opposed to their statements; put simply, local governments’ frequently say one thing while doing another.

Furthermore, although Indigenous identity is recognised, the articulation of Indigenous peoples’ inclusion within neoliberal economies endeavours to foreclose other alternative economic arrangements. While we do not, in this book, focus on economics, it is nevertheless important to acknowledge this significant critique of recognition-based justice. Scholars highlight the fundamental need for local arrangements that allow for Indigenous peoples to be agents of recognition thereby gaining control over the redistributive of revenues and expenditures directed at addressing Indigenous peoples’ socio-economic disadvantage and marginalisation, and in doing so promote Indigenous peoples’ inclusion and address injustices; this is how “recognition from below” takes place, “when people in dominated social positions turn away from institutionalised power hierarchies, shaping their own social orders without approval or permission of any authority beyond themselves” (Williams 2014, p. 10). As Williams observes, these “processes of the state self-constituting power”, realised through formal political movements or acts of resistance, also involve struggles for recognition, but the “agents of recognition” are Indigenous peoples rather than the state. Evidence of what Coulthard terms “recognition from below” which he defines as the: strategies of ‘self-recognition’ through which colonised or dominated subjects “critically revalu[e], reconstruct … and redeploy … culture and tradition” and, through such a process, transform their own subjectivities and consciousness as political agents (Coulthard 2007, p. 456). Significantly, many scholars examine the dynamic and complex trajectories of neoliberalism within settler-nations and highlight how neoliberal governance frequently involves a shift in state recognition of Indigenous interests and demonstrates what is needed to create situations where recognition from below is possible. For instance, Will Sanders argues (in the context of Australia but equally applicable to other settler-nations) that what is needed in contemporary Indigenous policymaking is some re-recognition of decolonisation as a means to address continuing Indigenous socio-economic disadvantage (Sanders 2018). He goes onto suggest that labelling and framing are significant, and it is critical to continue to insist articulating and acting on the process of decolonisation (even if we live in the age of neoliberalism) because it keeps alive the central ideas about the critical need to recognise Indigenous interests and demands for justice.

Such ideas can also be extended to thinking about IEJ as there are concerns that the state continues to be the arbiter decider of what and how Indigenous rights and interests in water (land, seas, and so forth) are recognised (as we demonstrate in Chap. 10.1007/978-3-030-61071-5_4). What this means, as we explore in-depth in Chap. 10.1007/978-3-030-61071-5_9 (which explores river restoration), is the nuances and complexities of Indigenous interests in their local environments, which includes their use of natural resources and environmental stewardship across successive generations as well as deliberative forms of place-based and kinship-centred governance, are frequently overlooked in favour of recognition formats that fit the needs (worldviews and governance structures) of the settler state rather than Indigenous peoples’ themselves. In doing so, the plethora of intergenerational environmental injustices experienced by Indigenous peoples is frequently overlooked by the narrow “recognition from above” models employed by the states. However, we demonstrate the potential to disrupt the narrow conceptualisations of recognition and extend it to include multiple ontologies and legal orders. We suggest that there is an emerging middle ground between a settler state and Indigenous political agendas in Aotearoa, which imperfect, in the context of the emergence of co-governance and co-management arrangements over rivers (and mountains) (outlined in Chaps. 10.1007/978-3-030-61071-5_7 and 10.1007/978-3-030-61071-5_8) does present the potentialities of addressing environmental injustice through governance structures and management approaches underpinned situated within Māori ways of knowing and beyond.

## Beyond Recognition: Indigenous Ontologies and Epistemologies

**Fig. 2.1:**
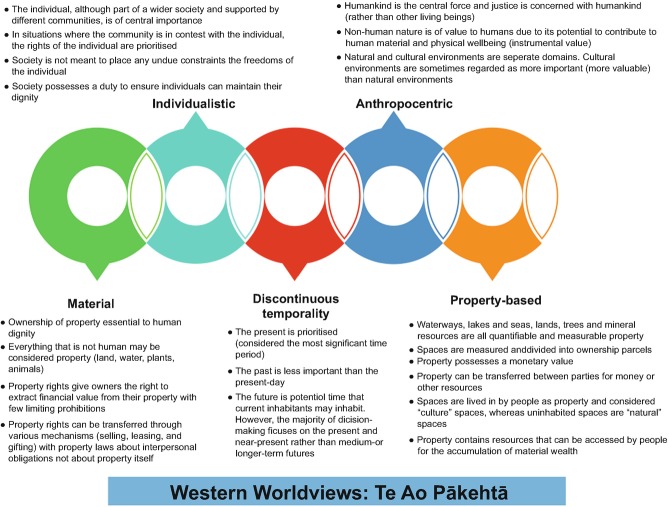
Key features of Western worldviews that pertain to discussions of EJ

**Fig. 2.2:**
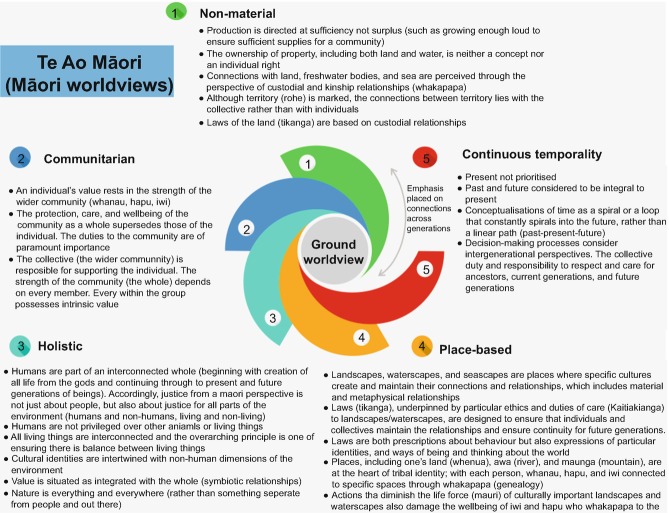
Key features of Indigenous worldviews

There is a fundamental need, Māori philosopher Christine Winter argues, for accounts of environmental justice to move beyond Western liberal thought to meaningfully include Indigenous ontologies and epistemologies (Winter 2018, 2019a, b). One way of doing this would be to expand the dimensions of recognitional justice to embrace ontological and epistemological pluralism. Winter identifies some of the differences between Indigenous and Western intellectual traditions (see Figs. 2.1 and 2.2) that inform our later discussions of EJ. Recent research by Indigenous scholars, including Winter, McGregor and Whyte, documents instances of environmental injustices suffered by Indigenous communities, which are tied to the continued dominance of Western worldviews (including framings of what constitutes justice as summarised in Fig. 2.1) that are premised on nature/culture binaries (already critiqued by a plethora of scholars) (McGregor 2018a; Todd 2016; Whyte 2018; Winter 2019a, b).

Despite how well-intended the EJ scholarship is, the dominant EJ framework being used by scholars (and applied to Indigenous communities around the globe) continues to neglect the unique experiences of Indigenous communities and their collective trauma under colonialism (Whyte 2016, 2017). A wealth of indigenous and non-indigenous academics call for the colonial structures that underpin EJ (as a movement and a field of study) to be overthrown to allow space to both acknowledge and enact the knowledge, rights, and sovereignty of indigenous peoples (Bird 1999; Gilio-Whitaker 2019; McCreary and Milligan 2018; Whyte 2016b, 2020). Indigenous scholars, in particular, argue that environmental issues facing Indigenous communities differ from those faced by non-Indigenous communities because of Indigenous cultures, identities, experiences of colonisation (including violence and dispossession), Indigenous knowledge systems, modes of life, and tribal sovereignty (Vickery and Hunter 2016). IEJs (IEJ) makes explicit the relationships between indigenous worldviews, cultural continuance, and sovereignty which all embody crucial components of power, authority, and justice within Indigenous contexts (Holifield et al. 2017; Weaver 1996, 2016; Whyte 2011). Because how environmental decision-making, both in the past and present-day, centres on only one way of relating to the environment, institutions develop particular ways of doing things over time which are underpinned by the idea that Indigenous environmental governance and management approaches are of marginal or no importance (Steel and Whyte 2012; Whyte 2018). Here, the lens of IEJ provides us with the opportunity to acknowledge both Indigenous sovereignty and indigenous worldviews as rooted in justice-oriented freshwater governance management and decision-making.

Māori worldviews, which exist on a continuum that is increasingly incorporating Western liberal individualism as well as Māori collectivism, continue to resonate in and shape Māori people’s lives and their engagement with their awa as we outline in later chapters. Like other Indigenous people who live within the borders of settler-colonial states, Māori iwi (tribes), hapū (sub-tribes), and whānau (extended family) endure despite the social, cultural, economic, political, and ecological marginalisation they experienced as a consequence of settler colonialism. Indeed, the histories of Māori and other Indigenous cultures over the last two hundred plus years of colonisation offers us all (Indigenous and non-Indigenous alike) essential lessons about what constitutes a life well-lived and how to maintain cultures, identities, a sense of belongingness and connectivity, and pursue a good life (one that holds value to you) in the face of radical (seemingly Earth-shattering) social, economic, political, cultural, and environmental changes. Indeed, the populations of Indigenous peoples around the globe experienced a massive loss of life as a consequence of infectious disease outbreaks linked to the arrival of colonisers bringing with them new diseases; smallpox, for instance, killed between 60–80 per cent of the Indigenous peoples of the Americas and Australia (far more than colonial violence ever did). While Indigenous peoples, like all peoples around the globe, are facing the COVID-19 pandemic, it is worth remembering the long history of past experiences of destruction and loss, and how different ways of thinking about the world can guide daily and future practices for more sustainable and hopeful futures. Indeed, Māori ontology can offer valuable learnings into two theoretical domains—dignity and time—that offer the potential to address both Western and Indigenous demands for EJ and intergenerational justice.

For more than a century, Indigenous worldviews and philosophies were frequently excluded or disparaged, deemed either primitive or a-theoretical by scholars, scientists, and decision-makers alike (Mills 2015; Buckinx et al. 2015; Tully 2000). Despite persistent attempts to erase, replace, and eradicate Indigenous beliefs and worldviews (be it through academia, the legal system, policymaking, media and the education curricula), such values and understandings remain relevant to the lives of many Indigenous peoples. Increasingly, as the emergent co-governance and co-management approaches attest to, settler states are enacting policies (after centuries and decades of protests and campaigns by Māori groups) that recognise Indigenous authority, knowledges and principles (which challenge the supposed universal applicability and superiority of Western liberal thought). These portrayals shape current lives and will affect future generations of Indigenous peoples (as individuals, communities, and societies). A new concept of intergenerational EJ could, however, include and encourage Indigenous and non-Indigenous alike.

Some Indigenous ontologies are characterised as holistic and kin-centric, such as found amongst Māori of Aotearoa, Aboriginal peoples of Australia, and Indigenous peoples throughout North and South America. People are active and co-producing (participatory) players within ecosystems (see Burarrwanga et al. 2013; etc.). Without the wrenching division between humans and nonhumans which characterises Western thought (post the European Enlightenment) (Ghosh 2018), Indigenous peoples exist in a complex and highly dynamic continuum of relationships with natures (physical, ecological and metaphysical). So interwoven are these connections that some scholars include places as co-authors (Country et al. 2016; Suchet-Pearson et al. 2013). As Australian Indigenous scholar Laklak Burarrwanga (an elder from Datiwuy located in North East Arnhem Land) and collaboration with Indigenous and non-Indigenous scholars writes:

our homeland of Bawaka as co-author. That’s because the land, the water, the animals, the plants, the rocks, the thought and songs that makeup Bawaka contribute to what we are saying here in important ways. They speak to us, inform what we do and have guided our thinking and talking). (Burarrwanga et al. 2013, p. Loc 324 of 3120)

Indeed, many Indigenous cultures are therefore located on such intellectual groundings, ontological underpinnings firmly rooted in the lack of distinction between human and nature. It is, therefore, a crucial counterpoint to the Western liberal dichotomy of human-nature, civilised-savage, tamed-wild, productive/wasteful, modern/primitive, from a holistic and connective perspective that situates people as part of nature: “Humans can no more go out of nature than they can go out of their bodies” (Green 2011, p. 132).

Accordingly, this raises several critical questions about freshwater management in the Anthropocene, both in terms of theorising about EJ and actions to address the drivers and implications of freshwater degradation. Western liberal theorises of EJ (which remain dominant within both the international scholarship and policymaking domains) continue to claim neutrally, impartially, and universally. Yet, Indigenous scholars, including Watene and Winter, are challenging the field of EJ to reconsider and extend what constitutes life and dignity supporting environments for all peoples around the globe (including those from non-Western cultures) in the context of changing climate conditions and its intergenerational justice ramifications (Budowle et al. 2019; Spiegel et al. 2020; Watene 2016; Winter 2019b). The critical question is, what does EJ look like if we are to take into account the ontologies of Indigenous peoples in the context of freshwater governance and management? Is it possible to formulate, within the Western liberal theories of justice, an account of EJ (incorporating social, environmental and intergenerational justice) that provides for Indigenous peoples within settler societies? Indeed, are Western liberal theories capable of doing this or are the ontological differences so significant that the conceptualisation of justice is different? We attempt to address some of these questions in the following chapters in this book.

Winter identifies the ways in which the dominant cultures of settler states remain epistemologically ignorant of Indigenous perspectives. It is not possible to describe in-depth all Indigenous worldviews, but we do identify some standard features that differ from those of Western worldviews: non-materiality; a sense of place; communitarianism; holism; and non-linear temporality (summarised in Fig. 2.1). Likewise, other scholars challenge Western articulations of EJ and advocate for Indigenous-informed EJ approaches. There is no agreeable definition of what exactly counts as IEJ; however, McGregor summarises the approach that advocates for “relationships based on environmental justice [that] are not limited to relations between people but consist of those among all beings of Creation” (McGregor 2010, p. 27). Indeed, a common feature of the various IEJ scholarship is a framing of EJ that goes beyond humans (the anthropocentric lens) to include animals, plants, weather, geology, spirits and supernatural beings, and IEJ thus deserves an Indigenous-informed framework (distinct from EJ frameworks employed in Canada, United States, Australia and elsewhere). IEJ as a framework, McGregor et al. (2020) argues, provides a set of logics that moves beyond the myopic anthropogenic lens of Western liberal theorising to recognise and include more-than-human actors as well as the Earth itself (McGregor 2018b; McGregor et al. 2020). For example, in the context of freshwater management and water justice, scholarship exploring Indigenous knowledges and experiences of water injustices highlight how, for many different Indigenous peoples, water is conceptualised as a living, more-than-human entity with responsibilities and duties to maintain the life and wellbeing of itself and other beings, which contrasts markedly from Western understandings of water as a resource and commodity (Jackson 2018; McGregor 2015; Perreault et al. 2012; Stensrud 2016). According to Indigenous ontologies, as we explore further in Chap. 10.1007/978-3-030-61071-5_6, issues of water justice and security are not merely about Indigenous peoples (and other social groups) being able to access water equitably (as encapsulated in the United Nations right to-water discourse) but also about justice for water as a more-than-human entity who possesses its own rights and responsibilities, which need to be recognised and provided for (Jackson 2018; McGregor et al. 2020).

In line with other Indigenous-informed approaches to EJ and maintaining the significance of EJ being spatial and temporally located (considering local histories, cultures, and geographies), in the rest of the book we explore Māori (specifically Ngāti Maniapoto) conceptualisations of and responses to environmental injustices. However, we do draw links to other Indigenous peoples’ ontologies and framings of justice (with particular emphasis on reciprocal relations, intergenerational responsibilities and more-than-human entities) to highlight the ways in which a growing chorus of EJ scholars and activists are drawing attention to other forms of knowing and being and the limitations of the hegemonic (Eurocentric) EJ paradigm. For instance, Māori emphasise the need to manage environmental resources sustainably (guided by the principle of kaitiakitanga meaning environmental guardianship) to ensure that future generations can use those resources (which we explore in future depth in Chap. 10.1007/978-3-030-61071-5_2). A commonly used whakataukī (proverb used within Māori societies to share cultural norms and values) that encapsulated the intergenerational dimension of Māori environmental management:

Hutia te rito o te harakeke. Kei hea te korimako, e ko? Ki mai ki ahau, he aha te mea nui o te ao? Maku e ki atu He tangata, he tangata, he tangata.

Pluck the heart from the flax bush - where will the bellbird be? Ask me, what is the most critical thing in the world? I will reply, it is people, it is people, it is people. (Cherrington 2019, p. 53)

While the meaning of this whakataukī is multi-layered, its central message is one of sustainability. It underpins the idea that balance is needed between all elements of the world (humans and more-than-humans) to maintain the health and wellbeing of all (Durie 2006; Rixecker and Tipene-Matua 2003; Walker et al. 2019; Wehi and Lord 2017). Harakeke (the flax bush *Phormium Tenax*) is a prodigious plant that grows throughout Aotearoa and is (and historically was) used for a variety of purposes by Māori (specifically for the weaving of clothing, art, baskets and ropes). Accordingly, efforts are taken to use it sustainably. For instance, the side leaves of a flax plant can be removed, but if the plant’s central core is damaged, the plant will die. Likewise, the korimako (bellbird *Anthornis Melanura*) collects nectar from the flowers of flax bush (and is also praised for its beautiful song). So, the death of flax negatively impacts the health of bellbirds. The answer to the question stresses that people must practice reciprocal relationships with the more-than-human beings that share the world(s) with them and emphasises the sustainable use of resources to ensure the wellbeing of current and future generations.

Far across the Pacific Ocean, in the Canadian context, Anishinaabe scholar Deborah McGregor articulates similar ideas in her research into Anishinaabe EJ. She demonstrates how, under Anishinaabe traditions, justice extends to include both current generations as well as the “ancestors of current beings and those yet to come (at least as far ahead as seven generations from now)” (McGregor 2010, p. 30). For the Anishinaabek people, environmental management decision-making is required to consider at least seven generations of beings (human and more-than-human). Such conceptualisations of looking seven generations into the future are likewise articulated in various Canadian and US Indigenous peoples’ declarations about their rights and responsibilities for their waters, including the Water Declaration of the Anishinabek, Mushegowuk, and Onkwehonwe (2008) and the Tribal and First Nations Great Lakes Water Accord (2004).

In Australian Aboriginal societies, and even longer intergenerational lens is applied to conceptualisations of EJ that reflect different conceptualisations of time (which challenges assumptions of linearity and forward-thinking). In Australia, Australian Aboriginal peoples’ occupation traces back more than 50,000 years and Aboriginal clans have been living in their ‘Country’ (traditional lands and waters) for 2000 generations (something that Western scientists only recently “discovered” but Aboriginal peoples already knew and recounted in their oral histories and traditions). Each Australian Aboriginal people and their specific Country, therefore, are co-constituted. In the words of Winter: “Together they have weathered ice ages, sea-level rise and fall, drought, and storms, extinctions and the flourishings: these changes are recorded in their stories” (2018, p. 127). Within Australian Aboriginal cultures, the land is the source of identity, and everything is interwoven back to and within reciprocal relationships with the land. The Aboriginal people come from the *country*, and they return to it where they reside as ancestors (underpinned by cosmological thinking of Dreamtime and Dreaming). There is more this understanding of reciprocal and intergenerational relationships. The ancestral beings, ( more-than-human beings who lived on the Australian continent before humans occupied the landmass), provided the form to the original human beings. That is, as Moreton-Robinson highlights, such ontological relationships centre on the connectivity of ancestral beings with the land and humans as co-constituted and interwoven embodied entities, wherein injustice against one is an injustice against all (Moreton-Robinson 2015, p. 12, 2017). As Aboriginal legal scholar Irene Watson writes:

The Nunga ‘I am’ is not like the other, dominant Western subject of being, which is represented by a straight line of thought—beginning, middle and ending. Instead, a Nunga process encircles; within there is a process that allows a person to become one and to begin again. This process is non-hierarchical and non-linear; rather it takes the form of a cycle, of the continuity of being, becoming another cycle, nurntikki [to go on forever]. (Watson 2014, p. 16)

As an Australian Aboriginal person comes from the land and ancestral beings come from the ground before returning to the land and living within the land, from where they (people/ancestors) may arise again in some other form. Accordingly, “when listening to *country* Aboriginal people listen to ancestors, bringing them into the present, including them within an intergenerational, inter-species, inter-form community” (Winter 2018, p. 129). Such listening is an active process wherein Aboriginal people narrated how their whole body is involved in listening. It requires them to interpret the results (what they hear) in light of their specific responsibilities to care for country and past/present/future generations of humans and ancestors (Maclean and The Bana Yarralji Bubu Inc. 2015; Moreton-Robinson 2015; Woodward and Marrfurra McTaggart 2019; Zurba and Berkes 2014). More in-depth understandings of Indigenous philosophies and justice theorising are provided by Deborah McGregor, Kyle Powys Winter and Christine Jill Winter. We offer here just a brief introduction to some of these and other scholars’ works to make it clear (in contrast to the Western framing of justice that emphasis universality) that Indigenous peoples can experience injustice differently (to non-Indigenous peoples and other Indigenous peoples). Furthermore, the types of actions that are (or should be) taken to address environmental injustices, therefore, need to take into account these differences (historical, biophysical, socio-cultural, economic, political, and ontological).

## Conclusion

In the following chapters of this book, we advocate for thinking about IEJ in intergenerational, pluralistic, and relational terms, which extends to include the material and metaphysical and does not institute strict divisions between humans and more-than-human actors, between land and water, or between past, present, and future generations. We argue and demonstrate how Ngāti Maniapoto environmental injustices were and are not extraordinary one-off events (a flood) or singular causes (a polluting factory) rather injustices build up over time. In this book, therefore, we explore how Māori challenges to settler-colonial governance and management of the Waipā River, along with other river systems in Aotearoa, are examples of Māori iwi and hapū rangatiratanga (chiefly authority) and their cultural continuance, despite their ongoing experiences of settler colonialism (invasion, dispossession, socio-economic and political marginalisation, attempts at cultural assimilation). The existing scholarship on IEJ indicates that the sophisticated practices of historical colonialism and political economy are evidence of indigenous communities’ around the globe’s ongoing struggles to maintain and re-assert their rights of self-determination. In this book, we argue, that it is not just a struggle over self-determination and the political economy but also a conflict between contrasting worldviews (or ways of thinking about the world—ontologies) and practices (ways of acting in the world—epistemologies) between the Western liberal worldview (Pākehā/White New Zealand) and Māori worldview, which were reflected in how each group conceptualised the nature of the problem, potential solutions, and on-the-ground actions. Furthermore, we demonstrate how, even when government policies were designed (on paper) to protect the environment and allow for Indigenous communities to participate in environmental decision-making, the settler-colonial governments often applied their policies in a way that encouraged environmental degradation and limited community participation and, in doing so, exacerbated Indigenous environmental injustices. The EJ framework, at present, does not sufficiently take into account the influence of settler colonialism on Indigenous peoples and recognise that settler-colonial rule exacerbates and/or causes environmental injustices for Indigenous peoples. Accordingly, we draw on decolonial theory to consider how theorising about IEJ can move beyond the western liberal EJ dogma to Indigenous ontologies and epistemologies (Álvarez and Coolsaet 2018; Barker and Pickerill 2019; Blaney and Tickner 2017; Davis and Todd 2017; Pulido and De Lara 2018; Rose 2004; Smith 1999). Whereas, the dominant framing of EJ (as a movement and body of scholarship) focuses on the human-to-human interactions with the environment as the background, IEJ, as we articulate throughout the rest of this book (from the perspective of three feminist Māori/Pākehā/Other hybrids from Aotearoa), includes the interactions between humans and more-than-humans (nonhumans) on a spiritual, cultural, and temporal level.
